# Supplementary material for: Development of the Spanish Version of Sniffin’s Sticks Olfactory Identification Test: Normative Data and Validity of Parallel Measures
Source: Brain Sci. 2021 Feb 10;11(2):216. doi: 10.3390/brainsci11020216 (PMC7916642; doi:10.3390/brainsci11020216)
Supplement: Supplementary file 1 [file brainsci-11-00216-s001.zip › brainsci-1094816-Supplementary/tableS1.pdf]

Table S1. Descriptive statistics of Free recall, Recognition and Subjetive intensity scores per Sex and Age

Mean (SD)	[20, 30)	[30, 40)	[40, 50)	[50, 60)	[60, 70)	[70, >70)	Overall
**Men**							
Free recall	3.6 (2.88)	5 (1.63)	3.5 (2.88)	3.36 (2.13)	2.03 (1.97)	2 (1.97)	2.71 (2.24)
Recognition	12.8 (1.92)	12.75 (1.23)	13.3 (1.83)	13.43 (1.39)	12.71 (1.71)	11.34 (2.89)	12.61 (2.17)
Subjective intensity	7.27 (1.46)	7.25 (0.85)	7.13 (1.47)	6.97 (1.4)	6.58 (1.17)	6.27 (1.14)	6.71 (1.29)
**Women**							
Free recall	2.5 (2.13)	4.11 (2.68)	3.53 (2.53)	3.55 (2.35)	3 (2.35)	2.25 (1.98)	3.13 (2.36)
Recognition	12.43 (2.1)	13.95 (1.54)	13.6 (1.54)	13.44 (1.66)	12.94 (1.96)	11.72 (2.98)	12.96 (2.18)
Subjective intensity	8 (1.14)	7.2 (1.15)	7.39 (0.98)	6.97 (1.33)	6.87 (1.38)	6.59 (1.27)	6.98 (1.31)

SD = standard deviation
