# Supplementary material for: Development of the Spanish Version of Sniffin’s Sticks Olfactory Identification Test: Normative Data and Validity of Parallel Measures
Source: Brain Sci. 2021 Feb 10;11(2):216. doi: 10.3390/brainsci11020216 (PMC7916642; doi:10.3390/brainsci11020216)
Supplement: Supplementary file 1 [file brainsci-11-00216-s001.zip › brainsci-1094816-Supplementary/tableS2.pdf]

Table S2. Tetrachoric correlation matrix of Blue version items from Study 2 sample

	Item 1	Item 2	Item 3	Item 4	Item 5	Item 6	Item 7	Item 8	Item 9	Item 10	Item 11	Item 12	Item 13	Item 14	Item 15
Item 2	0.205														
Item 3	0.501	0.291													
Item 4	0.671	-0.034	0.135												
Item 5	0.167	-0.179	0.063	0.101											
Item 6	0.227	-0.080	0.103	0.281	0.243										
Item 7	0.338	0.219	0.326	0.222	-0.037	0.139									
Item 8	-0.050	0.254	0.027	0.092	-0.049	0.039	0.053								
Item 9	0.426	0.387	0.424	0.072	0.184	0.081	0.314	-0.047							
Item 10	0.049	0.195	0.230	-0.311	0.067	0.014	0.209	0.019	0.220						
Item 11	0.315	0.077	0.223	0.526	-0.052	0.261	0.270	0.139	0.063	0.071					
Item 12	0.137	0.340	0.215	0.012	0.064	0.057	-0.069	0.273	0.199	0.254	0.099				
Item 13	0.523	0.074	0.358	0.554	-0.068	0.122	0.133	-0.005	0.117	0.289	0.207	0.142			
Item 14	0.339	0.253	0.422	0.298	-0.061	0.140	0.128	0.255	0.138	0.082	0.237	0.314	0.047		
Item 15	0.386	0.315	0.357	0.147	0.245	0.185	0.512	0.014	0.385	0.344	0.260	0.200	0.049	0.443	
Item 16	0.299	-0.098	0.334	0.319	0.038	0.235	0.119	0.114	0.377	0.268	0.687	0.450	0.169	0.222	0.218
