# Supplementary material for: Development of the Spanish Version of Sniffin’s Sticks Olfactory Identification Test: Normative Data and Validity of Parallel Measures
Source: Brain Sci. 2021 Feb 10;11(2):216. doi: 10.3390/brainsci11020216 (PMC7916642; doi:10.3390/brainsci11020216)
Supplement: Supplementary file 1 [file brainsci-11-00216-s001.zip › brainsci-1094816-Supplementary/tableS3.pdf]

Table S3. Tetrachoric correlation matrix of Purple version items from Study 2 sample

	Item 1	Item 2	Item 3	Item 4	Item 5	Item 6	Item 7	Item 8	Item 9	Item 10	Item 11	Item 12	Item 13	Item 14	Item 15
Item 2	0.256														
Item 3	0.156	0.107													
Item 4	0.058	0.119	0.145												
Item 5	0.090	0.020	0.034	0.157											
Item 6	0.050	0.212	-0.029	0.309	-0.176										
Item 7	0.264	0.022	0.378	0.573	-0.161	-0.006									
Item 8	0.207	0.223	-0.138	-0.052	-0.067	-0.093	-0.118								
Item 9	0.198	0.051	0.198	-0.098	0.024	0.233	-0.093	0.003							
Item 10	0.082	0.249	0.277	0.075	-0.031	0.171	0.289	0.049	0.056						
Item 11	-0.148	0.061	0.132	0.199	-0.008	0.177	0.136	-0.083	0.226	0.275					
Item 12	0.230	0.328	0.047	-0.108	0.014	0.028	0.270	0.206	0.243	0.267	0.176				
Item 13	0.350	0.319	0.247	0.112	0.047	-0.030	0.221	0.211	0.232	0.000	0.030	0.242			
Item 14	0.131	0.149	0.005	0.040	0.164	-0.002	0.179	0.111	0.017	0.007	0.241	-0.246	-0.049		
Item 15	0.150	0.070	-0.094	-0.154	-0.178	0.092	0.093	0.153	0.204	0.062	0.078	0.223	-0.052	0.049	
Item 16	0.055	0.182	0.151	0.069	0.079	0.033	-0.070	-0.038	0.059	0.165	-0.013	0.015	0.145	-0.053	0.227
